# Absence of CCR5 increases neutrophil recruitment in severe herpetic encephalitis

**DOI:** 10.1186/1471-2202-14-19

**Published:** 2013-02-07

**Authors:** Márcia Carvalho Vilela, Graciela Kunrath Lima, David Henrique Rodrigues, Norinne Lacerda-Queiroz, Vinicius Sousa Pietra Pedroso, Aline Silva Miranda, Milene Alvarenga Rachid, Erna Geessien Kroon, Marco Antônio Campos, Mauro Martins Teixeira, Johann Sellner, Antonio Lucio Teixeira

**Affiliations:** 1 Pós-Graduação em Ciências da Saúde: Infectologia e Medicina Tropical, Laboratório de Imunofarmacologia, Departamento de Bioquímica e Imunologia, Instituto de Ciências Biológicas (ICB), Universidade Federal de Minas Gerais (UFMG), Av. Antonio Carlos, 6627. Pampulha, Belo Horizonte, MG, 31270-901, Brazil; 2 Departamento de Biologia Animal, Universidade Federal de Viçosa, Viçosa, MG, Brazil; 3 Departamento de Microbiologia, ICB/UFMG, Belo Horizonte, MG, Brazil; 4 Centro de Pesquisas René Rachou, FIOCRUZ, Belo Horizonte, MG, Brazil; 5 Department of Neurology, Christian Doppler Klinik, Paracelsus Medical University, Salzburg, Austria; 6 Department of Neurology, Klinikum rechts der Isar, Technische Universität München, Munich, Germany

**Keywords:** Herpes simplex virus type 1, CCR5^-/-^, Neuroinflammation

## Abstract

**Background:**

The neuroinflammatory response aimed at clearance of herpes simplex virus-1 (HSV-1) plays a key role in the pathogenesis of neuroaxonal damage in herpetic encephalitis. Leukocytes activated in an adaptive immune response access brain tissue by passing through the blood–brain barrier. The chemokine CCL5/RANTES is involved in recruitment of these cells to the brain acting via the receptors CCR1, CCR3 and mainly CCR5. Here, we evaluated the role of CCR5 on traffic of leukocytes in the brain microvasculature, cellular and cytokines profile in a severe form of herpetic encephalitis.

**Results:**

Wild type and mice lacking CCR5 (CCR5^-/-^) were inoculated intracerebrally with 10^4^ PFU of neurotropic HSV-1. We evaluated the traffic of leukocytes in the brain microvasculature using intravital microscopy and the profile of cytokines by Enzyme-Linked Immunosorbent Assay at 1 day post infection. Flow cytometry and histopathological analyses were also carried out in brain tissue. Absence of CCR5 leads to lower viral load and an increased leukocyte adhesion in brain microvasculature, predominantly of neutrophils (CD11^+^ Ly6G^+^ cells). Moreover, there was a significant increase in the levels of MIP-1/CCL2, RANTES/CCL5, KC/CXCL1 and MIG/CXCL9 in the brain of infected CCR5^-/-^ mice.

**Conclusions:**

These results suggest that the absence of CCR5 may boost the immune response with a high neutrophil recruitment which most likely helps in viral clearance. Nonetheless, the elevated immune response may be detrimental to the host.

## Background

Herpes Simplex Virus (HSV) is a major cause of encephalitis in humans [1]. Conventional treatment of HSV-1 encephalitis (HSE) involves the use of antiviral therapies, current guidelines for the treatment of HSV-1 recommend intravenous acyclovir [2]. However, there is some evidence showing that modulation of inflammatory response may be beneficial [3]. In this regard, Kamei and coworkers [4] showed that higher proinflammatory cytokine levels in cerebrospinal fluid (CSF) from HSE patients were associated with a worse outcome of the disease and the decrease in cytokine levels after corticosteroid treatment resulted in a better outcome. Therefore the severity of the HSE depends at least in part on the inflammatory response [4].

The inflammatory response in the central nervous system (CNS) may lead to neuroaxonal damage. One of the characteristics of an inflammatory environment is the recruitment of leukocytes, which can pass through the blood–brain barrier. The recruitment of leukocytes is composed of a series of events which are dependent on multiple protein interactions [5-7]. Intravital microscopy studies allow the visualization of leukocyte/endothelial cell interaction in vessels *in vivo* and have revealed that leukocytes must first tether and roll along the venular wall before they can attach firmly and emigrate out of the vasculature [8,9].

Molecules called chemokines (chemotactic cytokines) are essential in the recruitment of leukocytes. Chemokines act mainly on the adhesion step of this highly regulated process, contributing to the migration process [9-11].

The chemokines CCL2/MCP-1, CCL3/MIP-1α, CCL5/RANTES and CXCL-8/IL-8, [12] are increased in the brain of patients with HSE. CCL5/RANTES is a chemokine member of CC family that recruits monocytes, T cells, basophils and eosinophils, acting via the receptors CCR1, CCR3 and CCR5 [13]. Chemokines constitute a rapidly growing family of proteins and receptor-chemokine interactions are known to be promiscuous and redundant. For instance, CCR5 is a G-protein-coupled seven-transmembrane receptor expressed in different cell types like T cells, macrophages, dendritic cells and microglia, and that binds to several chemokines, including: CCL3 (MIP-1alpha/Macrophage Inflammatory Protein b-chemokine), CCL4 (MIP- 1beta), CCL5 (RANTES), CCL8 (MCP-2), CCL11 (eotaxin), CCL14 (HCC-1), CCL16 (HCC-4) [14]. Besides the CC family of chemokines, there is also the CXC family of chemokines, which is important in the chemotaxis of several immune cells. For example, Kc/CXCL-1 and MIG/CXCL9 are chemokines that attract neutrophils and T cell to the inflammatory site, respectively. Kc presents a chemotactic role similar to that played by the human CXCL8.

Our research group has established a murine model of HSV-1 encephalitis in which intracranial inoculation of a neurotropic HSV-1 strain causes signs of encephalitis and death a few days after infection [15] in contrast with the nasal infection in which mice do not develop encephalitis [16]. We have also demonstrated that leukocyte rolling and adhesion in the brain microvasculature of HSV-1 infected mice was associated with the increased expression of chemokines in the brain [15].

In this experimental model, mice infected with HSV-1 and treated with a polyclonal anti-CCL5 antibody two hours before the intravital microscopy decreased leukocyte adhesion in the microcirculation [17]. These results led us to evaluate the specific role of CCR5 receptor, a receptor for CCL5/RANTES, in this model. CCR5 is constitutively expressed in astrocytes, microglia and neurons [18-20]. Its role in the healthy brain is still poorly understood, but CCR5 seems to regulate leukocyte maturation and trafficking in models of brain disease [21].

Here we investigated the effect of the lack of CCR5 in the trafficking of leukocytes, infiltration of immune cells, cerebral levels of cytokines/chemokines and viral load in a model of severe HSV-1 encephalitis.

## Results

### WT and CCR5^-/-^ infected animals present similar survival curves

**Figure 1:**
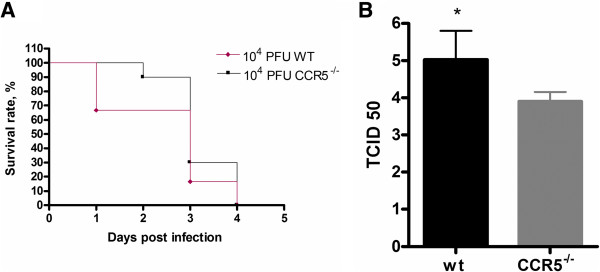
**Course of infection: WT and CCR5**^**-/- **^**mice were intracerebrally inoculated with 10**^**4 **^**PFU of virus HSV-1, and (A) survival was assessed daily.** (**B**) Viral load in brain at 1 dpi was assessed in WT (n = 6) and CCR5^-/-^ (n = 10). After sacrifice, brains were collected, macerated, and inoculated into Vero cell cultures to perform the titration procedure in triplicate. Survival curve was created using the product limit method of Kaplan and Meier and survival curves were compared using logrank test. Statistical analysis used to evaluate the viral load was Student’s *t*-test. Statistically significant results were indicated by ***p < 0.001, **p < 0.01 and *p < 0.05.

Wild-type (WT) C57BL/6 and CCR5^-/-^ mice were infected with 10^4^ PFU of neurotropic HSV-1. WT and CCR5^-/-^ animals presented similar survival curves, with 100% of mortality at 4 days post-infection (dpi) (Figure 1A). Using a non-lethal 10^2^ PFU *inoculum* all mice had an increase in survival rate (on average, six days more of survival), remaining no difference between WT and CCR5^-/-^ experimental groups. These results were confirmed in two independent experiments.

### CCR5^-/-^ infected animals present changes in viral load

Viral load in the brain was significantly lower in infected CCR5^-/-^ mice when compared to WT infected mice at 1dpi (Figure 1B).

### HSV-1 infection increased inflammatory cells, especially neutrophils, in the brain

FACS was used to determine the specific immune cell composition of brain infiltrates, targeting neutrophils, CD4^+^ and CD8^+^ T cells. Control mice had fewer neutrophils, CD4^+^ and CD8^+^ infiltrating cells compared to infected mice.

**Figure 2:**
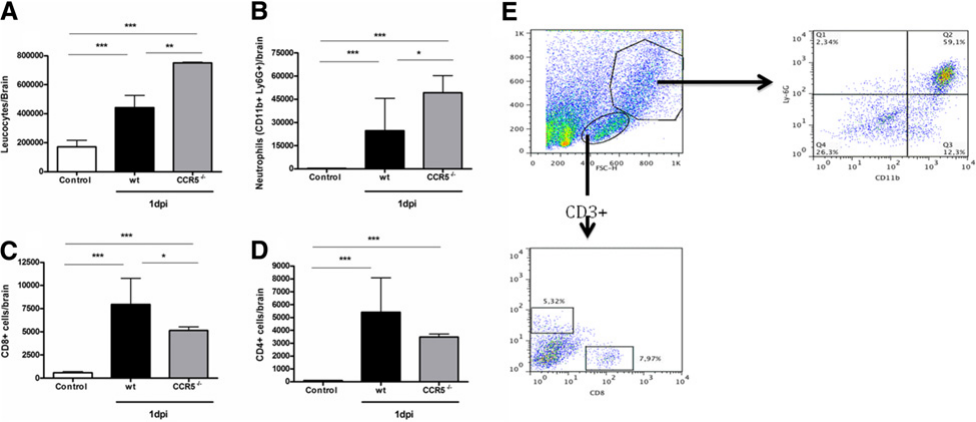
**WT and CCR5**^**-/- **^**mice were infected with 10**^**4 **^**PFU of HSV-1 by intracranial route and brain removed at 1dpi (n = 4).** Brain-sequestered cells were counted and then stained with specific antibodies. Neutrophils were characterized as CD11^+^ Ly6G^+^ cells and T lymphocytes were characterized as CD3^+^ TCD4^+^ or CD3^+^ TCD8^+^. Flow cytometry, according to size and granularity, was performed as analysis. Number of leukocyte (**A**), neutrophils (**B**), T lymphocytes CD8^+^ (**C**) and CD4^+^ (**D**) were evaluated in WT and CCR5^-/-^ mice. Results are expressed as mean ± SD and *p < 0.05, ***p < 0.001 when compared to non-infected mice. Representative gating strategy utilized for analysis of lymphocytes and granulocytes in the brain. Granulocytes population was isolated and collected for analysis. At this region, neutrophils were characterized as CD11^+^ Ly6G^+^ cells. Lymphocyte population was isolated and the cell population positive for CD3^+^ was collected for analysis. T lymphocytes were defined as CD3^+^ TCD4^+^ or CD3^+^ TCD8^+^. Flow cytometry data from a CCR5^-/-^ infected mice (**E**). Statistical analysis used: one-way ANOVA with Tukey correction.

The total number of immune cells in brain was significantly higher in CCR5^-/-^ mice when compared to infected WT mice. The brain of CCR5^-/-^ infected mice presented a significantly higher number of neutrophils, characterized as CD11^+^ Ly6G^+^ cells, compared with WT infected mice. The number of CD4^+^ T cells was similar in both CCR5^-/-^ and WT infected mice. Conversely the number of CD8^+^ T cells was higher in WT infected mice when compared with CCR5^-/-^ infected mice (Figure 2).

### CCR5^-/-^ infected mice present higher infiltration of leukocytes in the meninges

**Figure 3:**
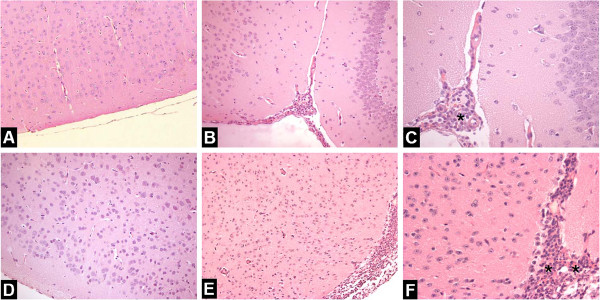
**Brain histopathological changes after intracerebral inoculation of 10**^**4 **^**PFU of HSV-1 in WT and CCR5**^**-/- **^**mice, at 1 dpi.** H&E-stained sections of meninges and cerebral cortex. Meninges of non-infected WT mouse (**A**) and CCR5^-/-^ animal (**D**) with normal brain tissue (n = 3 in each group); WT infected animal showing infiltration of immune cells in the meninges (**B-C**); Intense meningitis characterized by infiltration of polimorphonuclear and mononuclear in CCR5^-/-^ mouse (**E-F**). A-B: ×200; D-E: ×100; C-F: ×400.

Histophatological analysis was performed to indicate the localization of inflammatory cells. Brain slides from WT and CCR5^-/-^ control groups did not present histological changes. CCR5^-/-^ infected mice showed a marked increase in leukocyte infiltration in the meninges when compared with WT infected mice. Among inflammatory cells, polimorphonuclear cells, mainly neutrophils were the predominant inflammatory leukocytes found in the brain of CCR5^-/-^ infected mice (Figure 3).

### CCR5^-/-^ infected mice present higher leukocyte adhesion

**Figure 4:**
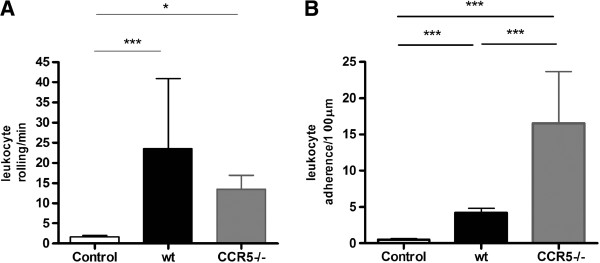
**Visualization of leukocyte-endothelium interaction at 1 day post infection with HSV-1.** WT (n = 6) and CCR5^-/-^ (n = 6) were intracerebrally inoculated with 10^4^ PFU of HSV-1. Intravital microscopy was used to assess rolling (**A**) and adhesion (**B**) of leukocytes on the brain microvasculature, at 1 dpi. Data indicate mean ± SD of cells per minutes (**A**) and 100 mm (**B**). Intravital microscopy revealed a significant increase in leukocyte adhesion in CCR5^-/-^ infected mice at 1 dpi (***p < 0.001). Statistical analysis used: one-way ANOVA with Tukey correction.

Intravital microscopy was done to visualize rolling and adhered leukocytes in meningeal vessels to understand the involvement of CCR5 receptor in the recruitment process. After 1 dpi, no differences in the number of rolling leukocytes were found between infected groups. Adhered leukocytes were significantly increased in infected CCR5^-/-^ mice when compared to infected WT mice (Figure 4).

### Cytokines and chemokines are increased in CCR5^-/-^ infected mice

**Figure 5:**
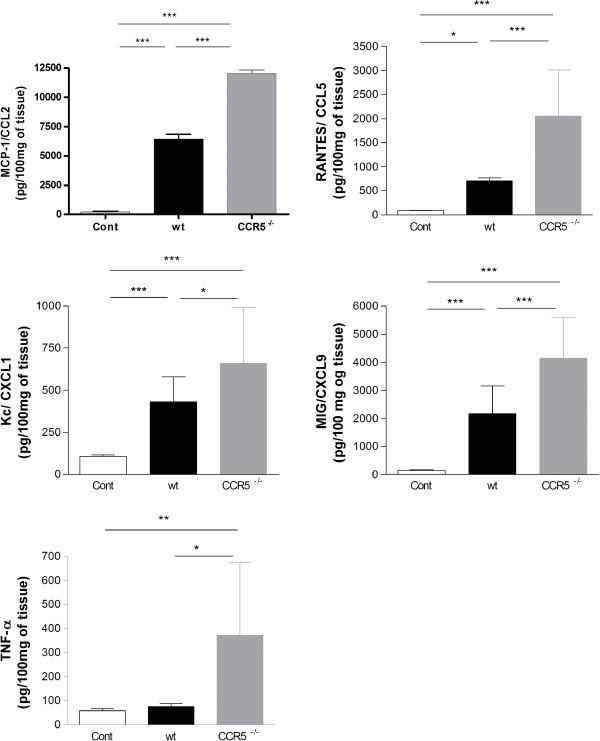
**Chemokine levels in the CNS after intracerebral inoculation with 10**^**4 **^**PFU of HSV-1 in WT (n = 10) and CCR5**^**-/- **^**mice (n = 13).** Cytokines were measured in brain extracts by ELISA at 1 dpi. The infection in CCR5^-/-^ mice was also followed by a significant increase in chemokine levels, including CCL2, CCL5, CXCL1 and CXCL9 and TNF-α level when compared with infected WT mice. Data indicate mean ± SD. Statistically significant results were indicated by ***p < 0.001, **p < 0.01 and *p < 0.05. Statistical analysis used: one-way ANOVA with Tukey correction.

After intravital microscopy, the brain was removed to evaluate the levels of the chemokines KC/CXCL1, MIG/CXCL9, MCP-1/CCL2, RANTES/CCL5 and the cytokine TNF-α by ELISA. At 1dpi, all chemokines and cytokine TNF-α were significantly increased in infected CCR5^-/-^ mice when compared to infected WT mice (Figure 5).

## Discussion

In the present work, we showed that CCR5^-/-^ mice infected with HSV-1 virus present an increased inflammatory cell infiltrate in the brain, composed mainly of neutrophils. In comparison with infected WT animals, CCR5^-/-^ mice also presented a reduction in HSV-1 load, while no changes were found in survival rate.

Previous studies have shown that C57BL/6 mice infected with HSV-1 strain EK did not develop encephalitis by peripheral (nasal) route [16]. Therefore, we standardized a model of infection by inoculating the virus HSV-1 strain EK by intracranial route. This route is associated with a severe disease characterized by diffuse meningoencephalitis in contrast with focal lesions determined by nasal or ocular inoculation [15].

The inoculum of 10^4^ PFU resulted in elevated rates of mortality in both WT and CCR5^-/-^ mice, suggesting that this viral load may be lethal regardless of the immune or inflammatory pathways involved. This prompted us to use a lower inoculum but the mortality rate of both groups remained similar. Interestingly, CCR5^-/-^ mice infected with HSV-1 virus by ocular route also showed no difference in mortality rate when compared to WT mice [22].

A lower HSV-1 load was observed in CCR5^-/-^ infected mice. This might suggest either that the virus presented a diminished replication rate or that the inflammatory response could be enhancing viral clearance in the brain. Both situations may be ascribed to the change in the type of cells infiltrating the brain. Histopathology and flow cytometry analysis showed an early increase in the number of total cells in the brain of CCR5^-/-^ mice, especially neutrophils. Neutrophils may contribute to virus clearance through the release of antiviral cytokines like TNF-α or oxygen and nitrogen reactive species [23].

Neutrophils are the first cells to infiltrate the cornea and they remain the predominant cell type during the development of HSV-1 keratitis [24-26]. This neutrophil response is known to both inhibit viral replication and induce corneal lesion [27,28]. In contrast with the present results, Carr and coworkers [22] found decreased neutrophil recruitment into the cornea and trigeminal ganglia in the early stage of HSV-1 infection by corneal route in CCR5^-/-^ mice. The number of recruited neutrophils in the cornea became similar in WT and CCR5^-/-^ mice only at day 7 p.i. Interestingly, there was increase in viral load in CCR5^-/-^ mice at day 7 p.i. which was probably secondary to defective neutrophil trafficking during the earlier stages of infection. At the same time, trigeminal ganglia and brainstems of CCR5^-/-^ mice revealed an increase of CD4 and/or CD8 T cells [22].

In the present work, there was also a significant change in leukocyte recruitment during HSV-1 brain infection. However, in contrast with a previous work [22], CCR5^-/-^ infected mice showed an increase of neutrophil recruitment in detriment of the CD4 and CD8 T cells recruitment in brain tissue. These conflicting results suggest that there is a significant difference between the immune response elicited in the periphery (i.e. cornea) and the response in the CNS in CCR5^-/-^ mice infected with HSV-1. When the virus is inoculated in peripheral sites, such as cornea, an immune response against HSV-1 can develop in trigeminal ganglia and related lymph nodes. This response can modulate the immune response before HSV-1 reaches the CNS, differing from the current study in which the virus was directly inoculated in the brain. Previous studies involving other *knock out* mice have also demonstrated conflicting data depending on the route of HSV-1 inoculation. For instance, Lundberg and coworkers [29] demonstrated that TNF-α antiviral effects are independent of TNFR1 in mice infected with HSV-1 by corneal scarification. When the virus was inoculated in the periphery of C57BL/6 mice, immune response of the host efficiently controlled virus infection even without TNFR1 [29]. Conversely, it was described a significant decrease in the survival rate of TNFR1^−/−^ mice infected by intracranial route [30]. The use of intracranial route is a way to elicit an immune response directly from the CNS, aiming to exclude immune activation in the periphery. In this regard, TNFR1 seemed to be relevant for the control of HSV-1 replication in CNS when there is no activation of the immune system in the periphery [30].

In contrast to neutrophil increase, the brain of CCR5^-/-^ infected mice showed a reduction of CD8^+^ cells. CCR5 is a member of the CC family of chemokine receptors that is expressed on a variety of leukocytes, including T cells and macrophages [31]. Accordingly, it would be expected that, without CCR5, recruitment of T cells might be reduced. The number of CD8^+^ T cells was also decreased in the brain of mouse hepatitis virus-infected CCR5^-/-^ mice as compared to WT mice [31].

Macrophages, CD4^+^ and CD8^+^ T cells are present in perivascular infiltrates close to and in contact with HSV-infected cells in areas of massive myelin loss necrosis in the brainstem of mice with HSV encephalitis [32]. CD8^+^ T lymphocytes are the first mononuclear cells that cross the blood brain barrier and move toward the infected tissue. The role played by CD8^+^ lymphocytes in HSV-1 encephalitis is still controversial. While one study reported no significant differences in viral load or viral reactivation when the virus was injected in cornea of mice lacking CD8^+^ T cells [33], another report showed persistent elevated viral titers in the brain of CD8-deficient, suggesting a role for CD8^+^ T cells in the control of HSV-1 replication [34]. In the current study, we found no difference in the levels of CD8^+^ lymphocytes in CCR5^-/-^ mice, thus, these cells cannot account for the decrease in viral load in the brain of CCR5^-/-^ mice. Following CD8^+^ T lymphocytes, tissue-penetrating Th1 CD4^+^ T cells contact local antigen presenting cells. This may result in an up-regulation of MHC molecules and secretion of more chemotactic molecules [35]. The inflammatory site during encephalitis involves the immune cells cited above and also B cells. Mice deficient for B cell had increased susceptibility to HSV-1 after peritoneal infection, presenting increased viral replication in the CNS. Thus, those results evidenced the importance of B cells in the control of viral replication.

Interestingly, treatment with anti-CCL5 or Met-RANTES, an antagonist of the CCL5 receptors CCR1 and CCR5, had no effect on viral titers but significantly decreased the number of leukocytes adherent to the pial microvasculature [17]. CCL5/RANTES is able to bind other chemokine receptors like CCR1 and CCR3, possibly compensating the absence of CCR5 in the trafficking and migration of leukocytes [36]. Using intravital microscopy, we found that CCR5^-/-^ infected mice presented an increase of leukocyte adhesion to the pial microvasculature. Bearing in mind that CCR5 is one of the most important receptors in leukocyte migration, this result seems paradoxical. However, one limitation of this technique is the impossibility to identify the kind of leukocyte that roll and adhere in vessels [8]. It is possible that those leukocytes observed in intravital microscopy are neutrophils.

Here we found an increase in cerebral chemokine levels, including KC/CXCL1, MIG/CXCL9, MCP-1/CCL2, RANTES/CCL5 and cytokine TNF-α in CCR5^-/-^ infected mice. Blocking the action of CCL5 receptor may result in compensatory mechanisms leading to the overproduction of other chemokines [37]. Thapa and coworkers [38] showed that mice deficient in CCR5 expressed higher levels of TNF-α, KC/CXCL1, MCP-1/CCL2, MIP1-α/CCL3 and RANTES/CCL5 in the brain after intravaginally HSV-2 infection. CCR5^-/-^ mice showed a significant elevation in chemokines MCP-1/CCL2, RANTES/CCL5, MIG/CXCL9 and IP10/CXCL10 in the trigeminal ganglion and brainstem after ocular HSV-1 infection [22].

The increase in chemokine concentration within the brain was also found in a study addressing the role of CXCR3 in HSV-1 infection. This receptor is expressed predominantly on CD4^+^ and CD8^+^ T cells, subsets of NK cells, and peripheral blood B cells. CXCR3^-/-^ mice infected with HSV-1 in the cornea had an increase in CCL5, CXCL10, and IFN-γ in the brainstem and IL-6 in the brain tissue. In addition, CXCR3^-/-^ infected mice exhibited high levels of chemokines which were coincident with high viral titers in the brainstem. The authors suggested that the absence of CXCR3 expression would suppress the capacity of T cells to respond to HSV-1 infection through limited trafficking to the trigeminal ganglia [39].

In our study, the lack of CCR5 was associated with lower viral load, implicating this receptor in viral multiplication. However, it is not clear whether the virus depends on this receptor to enhance viral multiplication or the immune system mounts a different response in its absence, resulting in more efficient viral control.

Our study has several limitations. We did not measure CCL-3, which is another ligand for CCR5 and could be related to HSV-1 infection. We did not evaluate natural killer cells as well. These cells may be relevant for the control of viral infection as they are putative effectors in the response against virus. Also, future experiments for the evaluation of viral titers and chemokine levels each day after infection are warranted. There is a possibility that compensatory mechanisms could eventually impact the immune-associated phenotype of CCR5^-/-^ mice. Nevertheless, no differences between non-infected WT and knock out mice were observed in the parameters assessed.

## Conclusions

In conclusion, the present study suggests that the absence of CCR5 is associated with an enhanced immune response against HSV-1, reducing CNS viral burden. However, the death rate is unchanged probably as a result of excessive inflammatory response. Future studies are necessary to further investigate the fining tune of the immune response in HSV-1 infection and, hence, to try to solve the puzzle involving both protection and lesion in the CNS.

## Methods

### Mouse strains

Male (age 6 to 10 weeks) C57BL/6 wild type (WT) andCCR5-deficient (CCR5^-/-^) mice, backcrossed to the C57BL/6 genetic background for 10 generations of mice, were used in these experiments. All mice were obtained from Animal Care Facilities of ICB-UFMG, Belo Horizonte – Minas Gerais – Brazil. The ethics committee of UFMG approved all experimental procedures used.

### Virus

HSV-1 strain EK [40] was allowed to multiply in Vero cells and maintained with minimal essential medium (GIBCO, USA) containing 5% fetal bovine serum (FBS) (GIBCO) and 25 μg/μL of ciprofloxacin (Cellofarm, Brazil) at 37°C in a 5% CO_2_ atmosphere. Virus was purified in sucrose gradient [41] and the titers were determined in Vero cells as previously described [42]. The virus titers obtained were 1.1 × 10^8^ PFU/mL for HSV-1.

### Vero cells

Vero cells were maintained in minimal essential medium (GIBCO, USA) supplemented with 5% heat-inactived Fetal Bovine Serum and antibiotics in 5% CO_2_ at 37°C. These cells were used for multiplication and titration of the virus.

### Infection with HSV-1

C57BL/6 and CCR5^-/-^ mice were anesthetized by intraperitoneal injection of a mixture of ketamine (150 mg/Kg) and xylazine (10 mg/Kg), and the inoculum of 10^2^ PFU and 10^4^ PFU of the purified HSV-1 resuspended in 10 μL of phosphate-buffered saline (PBS) were injected intracranial in the right side of sagittal suture at the level of the eyes to reach the frontal cortical regions. Control mice received PBS.

### Intravital microscopy

Intravital microscopy of the mouse brain microvasculature was carried out as routinely performed in our laboratory [13]. Briefly, mice were anesthetized by intraperitoneal injection of a mixture of ketamine (150 mg/Kg) and xylazine (10 mg/Kg) and the tail vein was cannulated for administration of fluorescent dyes. A craniotomy was performed using a high-speed drill (Beltec) and the dura mater was removed to expose the underlying pial vasculature. Throughout the experiment, mice were maintained at 37°C with a heating paid and the exposed brain was continuously superfused with artificial cerebrospinal fluid buffer, an ionic composition in mmol/L: NaCl 132, KCl 1.95, CaCl_2_ 1.71, MgCl_2_ 0.64, NaHCO_3_ 24.6, dextrose 3.71 and urea 6.7, pH 7.4, at 37°C.

Leukocytes were fluorescently labeled by intravenous administration of Rhodamine 6 G- Sigma (0.5 mg/kg body weight) and were observed using a microscope (Olympus B201, X20 objective lens, corresponding 100 μm of area) outfitted with a fluorescent light source (epi-illumination at 510-560 nm, using a 590 nm emission filter). A silicon-intensified camera mounted on the microscope projected image onto a monitor. The number of rolling and adherent leukocytes was determined offline during video playback analysis. Three vessels were analyzed for animal. Leukocytes were considered adherent to the venular endothelium if they remained stationary for a minimum of 30 seconds. Rolling leukocytes were defined as cells moving at a velocity lower than that of erythrocytes within a given vessel. Pial vessels with diameters ranging from 50 to 120 μm were used, as most adhesion occurred in vessels of these sizes. Because of the greater variability in size of these vessels (compared with that of other tissues studied using intravital microscopy), we expressed leukocyte adhesion as number of cells/100 μm.

### Histology

Animals were sacrificed by cervical dislocation and brains were quickly removed after intravital microscopy and preserved in 10% formalin. The 5 μm paraffin sections were stained with hematoxylin and eosin (H&E) and examined at the optical level with an Olympus Microscope. The description of cells was performed in a blind fashion by a single observer using image processing software (Kontron KS300 V. 2.0; Kontron Elektronik Gmbh, Germany). Digital images were acquired for documentation.

### ELISA of the proteins in the CNS

Brain tissue extracts were obtained from control and experimental mice after intravital microscopy, and the brain was stored at -20°C. Thereafter, the brain was homogenized in extraction solution (100 mg of tissue per 1 mL), containing: 0.4 M NaCl, 0.05% Tween 20, 0.5% BSA, 0.1 mM Phenil metil sulfonil fluoride, 0.1 mM benzethonium chloride, 10 mM EDTA and 20 KI aprotinin, using Ultra-Turrax. Brain homogenate was centrifuged at 3,000 g for 10 min at 4°C and the supernatants were colleted and stored at -20°C. The concentration of chemokines MCP-1/CCL2, RANTES/CCL5, KC/CXCL1 and MIG/CXCL9 and cytokine TNF-α was determined using ELISA.

The supernatants of brain extraction, at a 1:3 dilution in the 1% BSA in PBS, were assayed in an ELISA set-up using commercially available antibodies and the concentrations according to procedures supplied by manufacturer (R&D Systems, Minneapolis, MN and Pharmingen, USA).

### Flow cytometry and cell sorting

Mice were anesthetized and perfused intracardially with PBS to remove both circulating and non-adherent RBCs and leukocytes from the brain. Brains were removed and adherent leukocytes isolated using a previously described protocol with minor modifications (19). Each sample (n) correspond a pool of 2-3 mice brains. Briefly, the brains were collected and homogenized gently using the sterile glass tissue grinder in RPMI 1640 medium containing 5% FCS. Homogenates were passed through a nylon cell strainer (70 μm; Becton Dickinson and Company, Brazil) and cells centrifuged at 400 g for 10 minutes. The pellet was resuspended on a 35% Percoll gradient (Sigma-Aldrich) and this deposited kindly on a 70% Percoll gradient. After centrifugation (1,100 × g), the leukocytes were collected at the boundary layer, resuspended in fluorescence-activated cell sorting (FACS) buffer (PBS containing 1% FCS and 0.01% NaN_3_) and counted. Brain-sequestered cells were stained for extracellular molecular expression patterns using monoclonal antibodies (mAb) against mouse CD3e conjugated to phycoerythrin (PE) (BD Pharmingen San Diego, CA; clone 17A2), CD4 to fluorescein isothiocyante (FITC) (BD Pharmingen San Diego, CA; clone L3T4), CD8α conjugated to Peridinin Chlorophyll Protein Complex (PerCP) (BioLegend; clone 53-6.7) for Mix 1, and Ly-6 G conjugated to fluorescein isothiocyante (FITC) (eBioscience; clone RB6-8C5), CD11b conjugated to PE-Cy5 (BioLegend; clone M1/70) for Mix 2 and isotype controls (all from BD Pharmingen San Diego, CA). For each sample, 20,000 cells from the lymphocyte population were scored. The frequency of positive cells was analyzed using a gate that included lymphocytes and granulocytes. Limits for the quadrant markers were always set based on negative populations and isotype controls. Cells were acquired on a FACS Calibur flow cytometer (BD Biosciences) and analyzed using the FlowJo 7.5.3 software (TreeStar Inc.). Analysis in FlowJo software took into account size (forward light scatter) and granularity (side light scatter) of populations. Frequency in number of an analyzed population in front of total acquired events was used in the construction of graphs.

### Statistical analysis

Data are shown as mean ± SEM. The Kaplan-Meier test was employed to compare survival rates. One-way ANOVA with Tukey’s correction was used for multiple comparisons. Statistical significance was set at p < 0.05.

## Competing interests

The authors declare that they have no competing interests.

## Authors’ contributions

MCV carried out immunological assays, intravital microscopy analysis and drafted the manuscript. GKL was responsible for the inoculation and quantification of the virus. DHR, NLQ, VSPP, ASM participated in immunological assays and intravital microscopy analysis. MAR performed the histopathological analysis. MAC and EGK participated in the design and coordination of the study. JS, MMT contributed to draft the manuscript. ALT designed the study and was responsible for the interpretation of experiments and editing the manuscript. All authors have read and approved the final version of the manuscript.
